# Novel Y-Chromosome Long Non-Coding RNAs Expressed in Human Male CNS During Early Development

**DOI:** 10.3389/fgene.2019.00891

**Published:** 2019-09-24

**Authors:** Martin M. Johansson, Philipp Pottmeier, Pascalina Suciu, Tauseef Ahmad, Ammar Zaghlool, Jonatan Halvardson, Elisabeth Darj, Lars Feuk, Christiane Peuckert, Elena Jazin

**Affiliations:** ^1^Department of Organismal Biology, EBC, Uppsala University, Uppsala, Sweden; ^2^Department of Immunology, Genetics and Pathology, Science for Life Laboratory, Uppsala University, Uppsala, Sweden; ^3^Department of Women’s and Children’s Health, International Maternal and Child Health (IMCH), Uppsala University, Uppsala, Sweden; ^4^Department of Public Health and General Practice, Norwegian University of Science and Technology, Trondheim, Norway; ^5^Department of Molecular Biology, Stockholms University, Stockholm, Sweden

**Keywords:** sex differences, gene expression, X-chromosome, Y-chromosome, long non-coding RNA, RNA sequencing, human brain development, Pan troglodytes

## Abstract

Global microarray gene expression analyses previously demonstrated differences in female and male embryos during neurodevelopment. In particular, before sexual maturation of the gonads, the differences seem to concentrate on the expression of genes encoded on the X- and Y-chromosomes. To investigate genome-wide differences in expression during this early developmental window, we combined high-resolution RNA sequencing with qPCR to analyze brain samples from human embryos during the first trimester of development. Our analysis was tailored for maximum sensitivity to discover Y-chromosome gene expression, but at the same time, it was underpowered to detect X-inactivation escapees. Using this approach, we found that 5 out of 13 expressed gametolog pairs showed unbalanced gene dosage, and as a consequence, a male-biased expression. In addition, we found six novel non-annotated long non-coding RNAs on the Y-chromosome with conserved expression patterns in newborn chimpanzee. The tissue specific and time-restricted expression of these long non-coding RNAs strongly suggests important functions during central nervous system development in human males.

## Introduction

**Figure 1:**
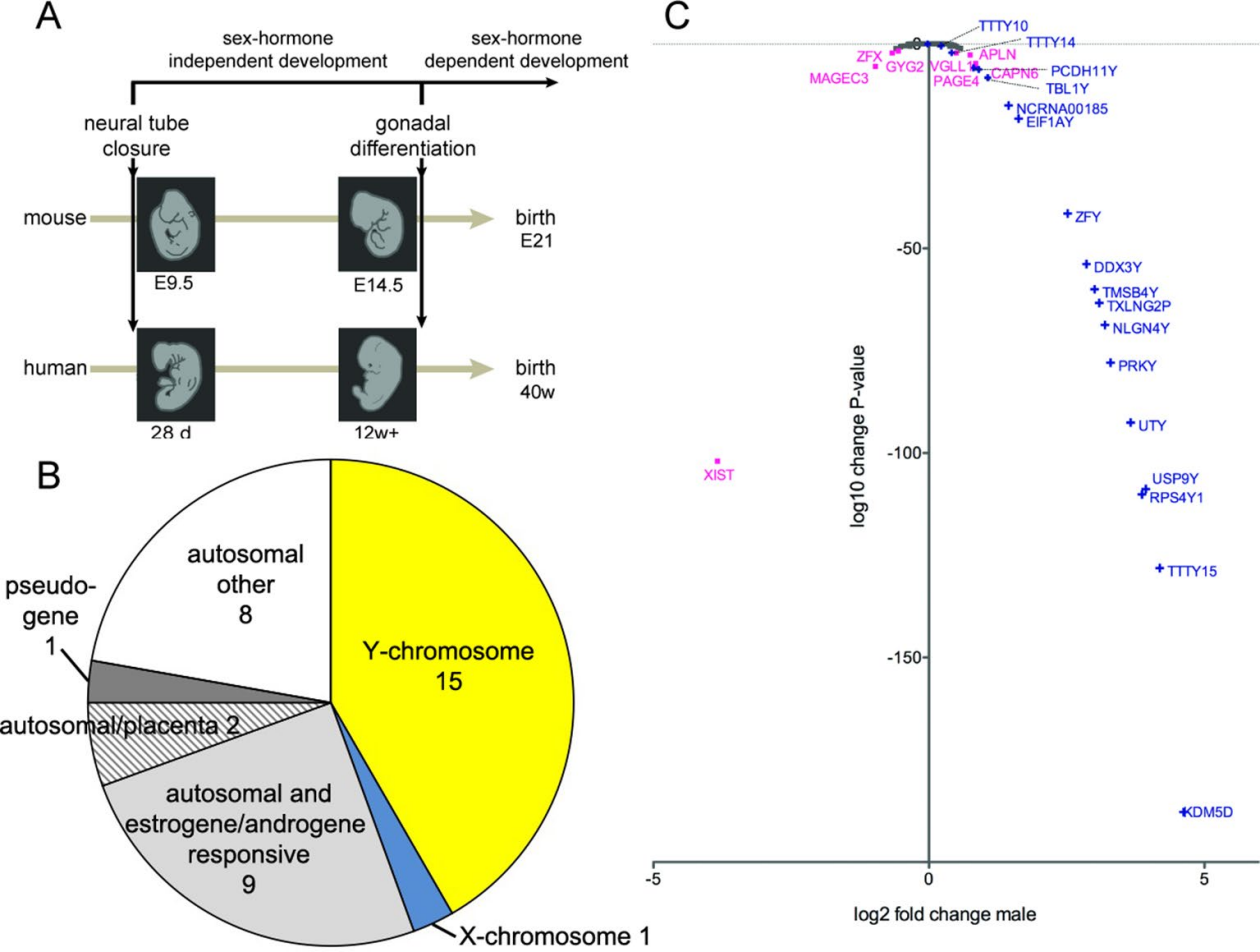
**(A)** Schematic timeline of development in mice (21 days) and in humans (40 weeks) until birth. The closure of the neural tube occurs at embryonal day 9.5 (E9.5) in mice and 28 days postgestation in humans. The primordial gonads start to produce sex hormones from E14.5 onwards in mice and from 12 weeks of gestation onwards in humans. **(B)** Classification of all genes that were differentially expressed in the medulla oblongata and midbrain of human male embryos during the first trimester, compared to female embryos. **(C)** Scatter plot of X- and Y-chromosome genes, showing log2-fold change of gene expression in male samples and corresponding log10-change P-values. Only genes with a change-P-value < 0.05 are labeled in color: Y-chromosome genes in blue and X-chromosome genes in purple; the gene names are indicated.

Gonadal hormones are powerful modulators of mammalian brain development and contribute to sexual dimorphism in organization and function of the nervous system. The first stages of the central nervous system (CNS) formation precede the maturation of the primordial gonads into ovaries or testicles that produce gonadal hormones (Arnold and Gorski, 1984; Gilbert and Barresi, 2016) (**Figure 1A**). In mice, the neural tube closure occurs at embryonic day 9.5 and from E14.5 onwards; testosterone is produced in male embryos (Pointis et al., 1980). In humans, the closure of the neural tube takes place about 4 weeks of gestation (wg) and represents a key event in early brain and spinal cord development. After approximately 12 wg, the circulation of the first estrogens or androgens can be detected (Reinisch, 1974; Resko, 1977). These events frame a period of very active CNS development in mammals, which is unaffected by sex hormones. During this sex-hormone-independent period, any genetically controlled gender bias in development should mainly be the result of the action of genes encoded on the X- and Y-chromosomes as well as the effect of imprinted genes (Arnold, 2004; Cahill, 2006; Jazin and Cahill, 2010; McCarthy and Arnold, 2011; Reinius and Kanduri, 2013). Indeed, in mice, several genes encoded on the sex chromosome are expressed in a sex-biased manner before sexual maturation of the gonads (Xu et al., 2002; Dewing et al., 2003; Xu and Disteche, 2006). In two earlier studies, where we analyzed more than 700 microarray expression datasets, we found that, in mice, only about a dozen of X- and Y-chromosome genes, respectively, have a significant expression bias between the sexes (Reinius et al., 2010; Reinius et al., 2012). In a previous study on human material, we described similar results showing that 11 Y-encoded genes were expressed in the brain of human male fetuses during the second trimester (Reinius and Jazin, 2009). These results were later supported by a study from Kang et al., where 9 X-encoded genes with sex bias and 13 Y-encoded genes were reported as expressed in males during the second trimester (Kang et al., 2011). Studies on the human brain during the first trimester are rare, due to the limited availability of samples. In this study, we report sex-biased expression in human CNS samples during the first trimester. Using high-resolution RNA sequencing combined with qPCR, we found that the Y-chromosome genes, described by Reinius and Jazin as well as Kang et al., are also present in this critical period of human development. In addition, we have found a group of newly discovered long non-coding RNAs that are expressed during the first trimester in males. We further investigated brain samples from male and female newborn chimpanzees, to evaluate whether the observed sex biases in humans are conserved during primate evolution.

## Results

### RNA Sequencing of Medulla Oblongata and Midbrain Samples From Female and Male Human Embryos During the First Trimester of Development

**Figure 2:**
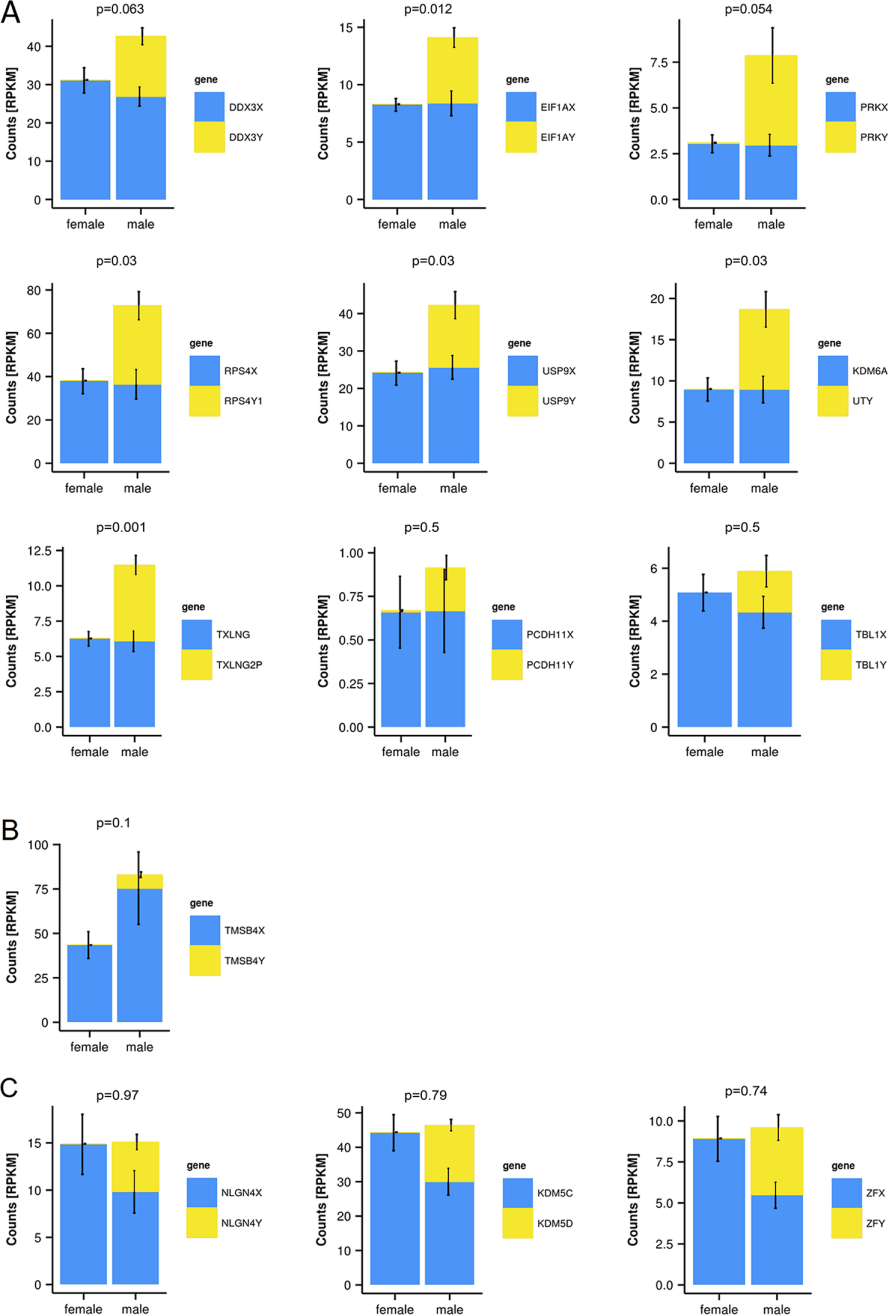
Y-chromosome-encoded gametologs display multiple features of sex differences based on gene dosage. RNA-sequencing data from female and male embryo samples, displayed as RPKM values, p-values for total gene-dosage on top of each graph (mean ± SD, n = 2, in triplicates from each total and poly(A) RNA). **(A)** Genes showing an increased dosage in male based on the Y-chromosome-encoded gametologs. **(B)** Increase of gene dosage in male due to X-chromosome-encoded gametologs. **(C)** Genes featuring dosage compensation.

To quantify expressed transcripts during CNS development in humans with high resolution, we performed RNA sequencing of samples derived from 2 female and 2 male embryos during the first trimester of development (**Table 1**). To increase the statistical validity, and because of the scarcity of samples from early embryos, we compared total RNA and poly(A) RNA from two regions (midbrain and medulla). After adjusting for multiple comparisons, only genes with a change p-value < 0.05 in both tissues were included in the final analysis. We selected only genes altered in both tissues to avoid a large number of false positives in each tissue. Of the 36 significantly differentially expressed genes (**Figures 1B, C** and **Table 2**), 15 were Y-encoded, 1 (*XIST*) was encoded on the X-chromosome, and 22 were autosomal genes. Nine of the autosomal genes could be estrogen-responsive, androgen-responsive, or responsive to both sex hormones (**Table 2**). Six of the autosomal genes (*ANGPT2, BHLHE40, MAFF, APLNR, SPON2, NLRP2*) code for proteins that play a role in developmental processes (*ANGPT2, SPON2, MAFF, APLNR*) bind to DNA (*SPON2, BHLHE40*) or are involved in innate immunity (*SPON2* and *NLRP2*) (Huang et al., 2009). Of the remaining genes, one is annotated as a pseudogene (*LOC644172*), and two genes (*PLAC4*, *PAEP*) are known to be highly expressed in placenta (Su et al., 2004). Not surprisingly, we only detected *XIST* among the genes coded on the X-chromosome. This is due to the scarcity of samples combined with the relatively small (1.2–2-fold) expression differences between sexes for genes that escape X inactivation (Xi), making our analysis underpowered to detect Xi-escapees. To increase the power for the detection of differentially expressed X-chromosome genes, we decided to analyze X- and Y-encoded genes in more detail using a slightly modified algorithm. Reads with a quality of 30 or higher were counted to avoid reads mapping to more than one genomic locations. Most RNA-Seq analysis count reads with a quality of 20 or higher (Sheng et al., 2017). Although this method would be more sensitive than ours having better chances of detecting larger number of genes, the problem of inflating results for gametologous pairs (due to high sequence identity between X/Y pairs) would still persist (see Materials and Methods). Of all 992 X-annotated genes, we found 8 genes (*PAGE4, MAGEC3, CAPN6, APLN, ZFX, VGLL1, GYG2, and XIST*) to be differentially expressed, of which 5 were known Xi-escapees (*MAGEC3, XIST, ZFX, VGLL1, GYG2*) (Tukiainen et al., 2017) (**Figure 1C** and **Supplementary Table 1**). From a recently published catalogue containing genes with indications to escape Xi in adult tissue (Tukiainen et al., 2017), we detected ca. 90% as expressed albeit without sex bias, except of the five abovementioned genes (**Supplementary Table 2**). These discrepancies can be explained in different ways. First, the Tukiainen study was performed on combined datasets from adult samples and various tissues, which makes a direct comparison difficult, especially since some Xi-escapees may not escape early during development. Second, as indicated above, our study is underpowered to detect Xi-escapees. Third, some previously described Xi-escapees may stem from inflated results due to expression of X-located pseudogenes with high sequence similarities. This would be observed if less stringent analysis (in terms of how RNA-Seq fragments are aligned to the genome) than the one performed here would have been used. In future, techniques such as RNA-FISH would be required to resolve the exact number of X-escapees, both during development and in adults (Reinius et al., 2012). Out of 125 transcripts on the Y-chromosome—which was the main focus of our study—15 were detected as expressed in males, including 13 with gametologs on the X-chromosome: *KDM5D/C, DDX3X/Y, EIF1AX/Y, PRKX/Y, TXLNG/P2, NLGN4X/Y, RPS4X/Y1, TMSB4X/Y, ZFX/Y, USP9X/Y, UTX(KDM6A)/Y, TBL1X/Y*, and *PCDH11X/Y* (**Figure 1C** and **Supplementary Table 2**). However, only *ZFX* was significantly biased in females. We then combined the gene expression levels for each gametolog in males to evaluate the effect of gene dosage. In our stringent RNA-Seq analysis, we excluded reads with 100% sequence identity between X and Y gametologous genes. In seven cases, the expression of the X-gametologs was very similar in both sexes. Therefore, the expression of the Y-gametologs contributed to a higher total expression of those genes in males (**Figure 2A**, p < 0.05 for 5 gametolog pairs). For *PCDH11* and *TBL1*, the expression values were low (<5 RPKM counts), and the differences in gene dosage were not significant. Our analysis may not have been sensitive enough to detect a significant difference. Also, *TMSB4* had a higher total expression in males, but the difference was not significant (**Figure 2B**). For *KDM5C, ZFX*, and *NLGN4X*, the expression was higher in females indicating escape from X inactivation. However, the total expression was balanced by the Y-gametolog expression in males (**Figure 2C**).

**Table 1:** Sample Information (w, week; d, day; MB, midbrain; MO, medulla oblongata; n, number).

n	ID	Age	Sex	Tissue	Experiment
1	E49	11w 2d— 1^st^ trimester	Female	MB	RNA-Seq
1	E54	11w 6d— 1^st^ trimester	Male	MB	RNA-Seq
1	E57	10w 2d— 1^st^ trimester	Female	MO	RNA-Seq
1	E55	10w 2d— 1^st^ trimester	Male	MO	RNA-Seq
1	E27	11w 0d— 1^st^ trimester	Female	MO	q-PCR
1	E65	9w 5d— 1^st^ trimester	Male	MO	q-PCR
4	636530	21–29 years old	Male	Whole brain	q-PCR
8	636533	24–87 years old	Male	Testis	q-PCR
59	636526	2^nd^ +3^rd^ trimester	Male/Female	Whole brain	q-PCR
1	Chimp	Neonatal	Female	Cortex	RNA-Seq, q-PCR
1	Chimp	Neonatal	Male	Cortex	RNA-Seq, q-PCR

**Table 2:** RNA- sequencing results.

Gene	Chr	Comment	Ref	Medulla					Midbrain				
				Base mean		Log2-fold change*	Change p-value	Adjusted change p-value	Base mean		Log2-fold change*	Change p-value	Adjusted change p-value
				Male	Female				Male	Female			
CGA	6	E-responsive	A)	21904	65,73	6,47	2,02E−204	3,65E−200	429,95	84,8	2,04	2,27E−08	1,94E−06
KDM5D	Y			2878,43	31,32	4,57	1,54E−85	1,39E−81	3829,51	27,88	6,52	1,47E−111	1,23E−107
PLAC4	21	Placenta	B)	3400,14	10,42	4,39	1,22E−66	7,33E−63	164,74	15,04	2,18	2,48E−05	0,001
DDX3Y	Y			2494,88	21,17	3,7	5,54E−47	2,00E−43	3016,95	19,27	6,32	2,15E−70	7,19E−67
EIF1AY	Y			253,33	1,66	3,06	2,72E−31	3,77E−28	326,32	2,99	5,04	3,72E−30	2,22E−27
PRKY	Y			629,41	12,39	2,82	5,16E−27	6,20E−24	1874,94	14,88	5,59	4,90E−41	4,55E−38
TXLNG2P	Y			875,64	5,87	2,82	1,72E−26	1,93E−23	1105,42	4,37	6,59	1,79E−67	4,27E−64
XIST	X			1297,18	144669	−2,75	3,43E−25	3,26E−22	1389,19	126612	−5,5	3,87E−46	5,39E−43
TTTY15	Y			2590,52	16,09	2,72	1,18E−24	1,06E−21	1986,42	14,22	6,19	9,51E−70	2,65E−66
S100P	4	E-and A-responsive	C)	303,26	6,85	2,61	4,30E−23	3,37E−20	43,44	5,26	1,62	0,003	0,048
NLGN4Y	Y			1497,49	15,06	2,21	5,62E−17	3,27E−14	1001,67	12,52	5,18	4,27E−38	3,40E−35
PAEP	9	Placenta	B)	208,9	3,47	2,2	1,18E−16	6,43E−14	240,96	9,1	3,88	1,67E−21	6,19E−19
RPS4Y1	Y			989,91	9,71	2,17	1,97E−16	1,05E−13	1420,23	9,05	5,55	2,20E−36	1,67E−33
SERPINE1	7	E-and A-responsive	D)	771,23	58,08	2,08	1,92E−15	9,91E−13	228,62	61,27	1,53	3,55E−04	0,01
FLT1	13	E-responsive	C)	4481,61	669,14	1,91	3,13E−15	1,57E−12	1271,94	382,9	1,53	7,49E−06	3,65E−04
TMSB4Y	Y			612,9	3,86	2,07	3,22E−15	1,57E−12	341,1	5,48	4,06	5,89E−17	1,61E−14
ZFY	Y			982,95	5,72	2,03	1,16E−14	5,36E−12	751,31	6,05	5,6	7,53E−43	8,39E−40
NCRNA00185	Y			288,15	2,29	1,89	4,65E−13	1,91E−10	200,33	2,01	3,88	3,12E−14	6,51E−12
ADM	11	E-responsive	E)	552,1	53,66	1,87	9,30E−13	3,65E−10	262,06	51,68	1,99	2,90E−07	1,98E−05
NLRP2	19			242,5	1066,89	−1,5	6,45E−10	2,04E−07	819,24	466,01	0,77	0,003	0,051
USP9Y	Y			7564,81	44,12	1,56	8,31E−10	2,58E−07	5004,53	35,22	5,38	9,09E−33	6,32E−30
H19	11			4158,38	110,38	1,55	1,87E−09	5,53E−07	724,99	299,76	1,08	0,006	0,09
ANGPT2	8			693,58	155,36	1,4	2,66E−08	6,40E−06	411,44	126,25	1,46	1,07E−04	0,004
TTTY14	Y			65,28	0,31	1,36	5,70E−08	1,28E−05	46,05	1,04	2,4	8,98E−06	4,25E−04
DPP4	2	E- and A-responsive	D)	66,11	5,21	1,16	6,39E−06	0,001	186,93	86,7	0,99	0,003	0,054
UTY	Y			2587,74	17,2	1,06	9,46E−06	0,001	3639,48	18,84	5,46	6,78E−08	6,82E−29
LOC644172	17	Pseudogene		0,62	23,61	−1,09	7,91E−06	0,001	1,53	61,74	−2,85	1,03E−31	5,37E−06
TBL1Y	Y			27,74	1,04	1,07	1,35E−05	0,002	239,76	0,33	5,37	5,45E−29	3,04E−26
COL3A1	2	E-responsive	D)	5828,57	1118,9	1,04	8,29E−05	0,01	707,72	7762,59	−2,84	5,20E−12	8,12E−10
FOS	14	E-responsive	D)	1289,81	467,11	0,96	1,27E−04	0,015	714,2	2799,12	−1,8	3,05E−09	3,17E−07
APLNR	11			304,62	747,88	−0,92	1,40E−04	0,017	123,81	1011,91	−2,63	6,01E−13	1,04E−10
IL8/CXCL8	4	E- and A-responsive	D)	30,12	0,94	0,88	1,62E−04	0,019	84,64	11,13	2,01	5,00E−05	0,002
MAFF	22			332,99	90,65	0,99	1,82E−04	0,02	250,54	51,44	2	1,86E−08	1,65E−06
SPON2	4			474,07	82,46	0,97	2,03E−04	0,022	35,11	194,75	−1,95	8,88E−06	4,24E−04
BHLHE40	3			970,92	352,66	0,9	4,41E−04	0,043	865,49	261,83	1,6	1,06E−08	1,01E−06
LYVE1	11			51,3	5,48	0,86	4,75E−04	0,046	83,35	220,69	−1,2	0,002	0,032

*Genes with significant different expression in male/female human embryo (E, estrogen; A, androgen).*

*The fold change is calculated as described in Materials and Methods using DESeq2, where a more complex definition than the simple ratio is utilized.

References for commented genes: A) Bieche et al., 2001, B) Su et al., 2004, C) Gerstein et al., 2012, D) Lachmann et al., 2010, E) Giacalone et al., 2003.

### Discovery of Novel Non-Annotated Genes on the Y-Chromosome

**Figure 3:**
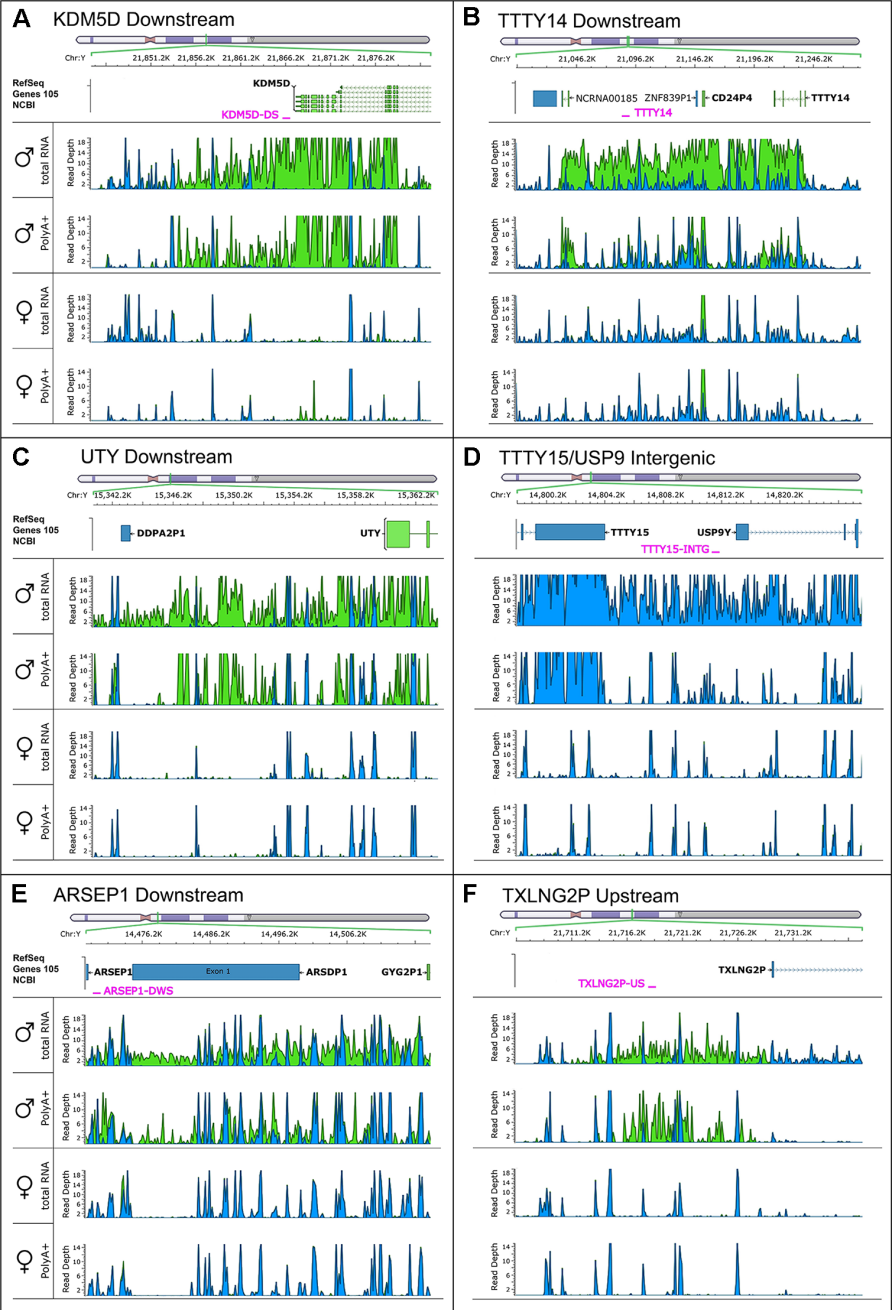
RNA-sequencing tracks indicate novel long non-coding RNAs on the Y-chromosome. Each subfigure shows a region in which RNA-Seq revealed a high read depth in a largely nonannotated area: *KDM5D* downstream **(A)**, *TTTY14* downstream **(B)**, *UTY* downstream **(C)**, *TTTY15/USP9* intergenic **(D)**, *ARSEP1* downstream **(E)**, and *TXLNG2P* upstream **(F)**. Four RNA-Seq tracks are displaying the total, as well as the poly(A)+ reads for male and female medulla samples. Blue color indicates reads on the positive strand; green color indicates reads on the negative strand. The name and position of qPCR primers are marked in red.

**Figure 4:**
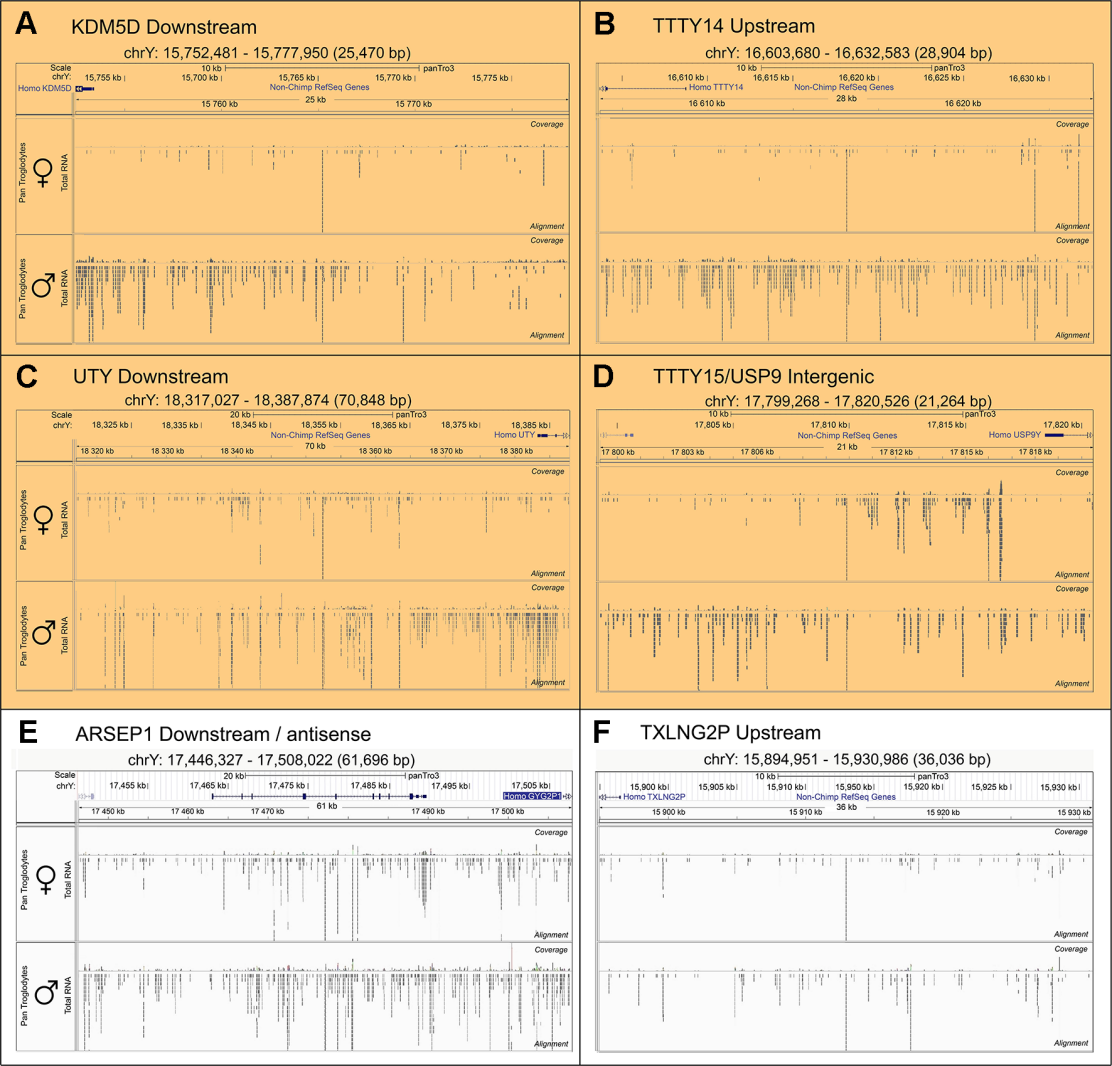
Conservation of RNA expression for novel Y-chromosome non-coding RNAs in chimpanzee. Sequencing tracks from newborn chimpanzee brain for the regions homologous to the human equivalents to **(A)** *KDM5D* downstream, **(B)** *TTTY14* downstream, **(C)** *UTY* downstream, **(D)** *TTTY15/USP9Y* intergenic, **(E)** *ARSEP1* downstream/antisense, **(F)** *TXLNG2P* upstream. The top segment of each sub-figure shows the position of the respective genomic segments on the chimpanzee Y-chromosome and the last exons of annotated genes located in proximity. The lower part of each sub-figure shows sequencing tracks for brain samples from a female (top) and male (bottom) newborn chimpanzee. The highlighted subfigures **(A**–**D)** indicate the RNA-Seq tracks that show YlncRNA expression exclusively in male chimpanzee samples.

To search for novel non-annotated genes encoded on the Y-chromosome, we visually scanned all our RNA-Seq tracks of total and poly(A) RNA samples for regions with high expression. We found six expressed regions in non-annotated sequence areas of the Y-chromosome (**Figure 3**). **Figure 3** also shows some signals in females. These result from misaligned sequences during RNA-Seq analysis, unavoidable due to repetitive elements in the genome included in these regions as well as other expressed regions throughout the genome. In our calculations, we considered these signals as the background level of the RNA-Seq analysis. Since no genes are annotated in these regions, they most probably correspond to long non-coding RNAs. These RNAs include a region of 22.5-kb (position 21,844,705–21,867,250 according to hg19 (Lander et al., 2001) downstream from the *KDM5D* gene (**Figure 3A**), a region of 14.3-kb (position 21,714,971–21,729,272) upstream and antisense from the *TXLNG2P* gene, a large region of 50.6-kb (14,467,868–14,518,442) downstream and antisense from the *ARSEP1* gene, a segment of 23.2-kb (15,337,148–15,360,300) downstream from the *UTY* gene; the longest transcribed region that was 182.4-kb (21,033,832–21,213,188) downstream from the *TTTY14* gene, and the 9-kb intergenic region between *TTTY15* and *USP9Y* (14,804,103–14,813,119). All of the above described regions except for the intergenic region between *TTTY15* and *USP9Y* indicate expression from the antisense strand. To evaluate whether these long RNAs may be functional, several criteria can be used (Palazzo and Lee, 2015). One criterion is the presence of polyadenylation signals. Three of the transcription signatures were present in poly(A) samples (**Figure 3**). The *TTTY15/USP9* intergenic transcript had similar poly(A) reads in male and female samples; however, the total RNA signals differed, suggesting the existence of a male specific RNA that might not be polyadenylated. A second criterion is sequence conservation in expressed RNAs between species. Using RNA sequencing on one newborn male and one female chimpanzees brain sample (**Figure 1**), we found four of the six regions, including *KDM5D* downstream, *TTTY14* downstream, *UTY* downstream, and *TTTY15* downstream expressed (**Figure 4**, **highlighted subfigures**).

### Expression of the Long Non-Coding RNAs Is Higher in Male Embryos Compared to Adults

**Figure 5:**
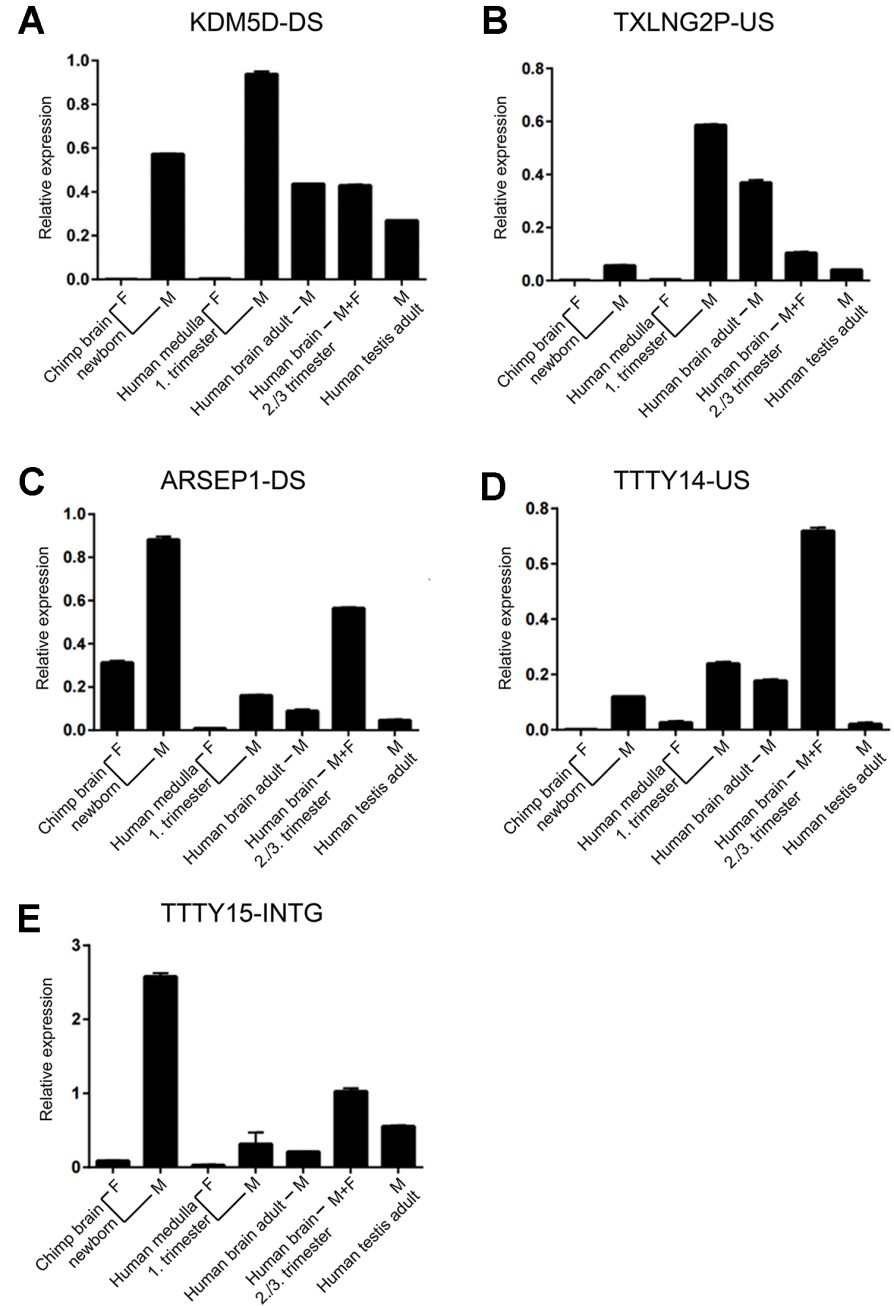
Expression of novel Y-chromosome non-coding RNAs. **(A)** KDMD-DS, **(B)** TXLNG2P-US, **(C)** ARSEP1-DS, **(D)** TTTY14-US, **(E)** TTTY15-INTG. Relative qPCR expression values (mean ± SD; normalized to *GAPDH* expression) for samples from brain of newborn female and male chimpanzee (n = 1 each), fetal human female and male medulla (first trimester, n = 1 each), human adult male brain (n = 4; pooled), a mix of human male and female fetal brain samples during the third trimester (n = 21; pooled), and human adult testes (n = 5; pooled). Each sample was assessed in duplicates. The names of the non-coding RNAs are indicated on top of each graph.

Of the six regions described above, in the *UTY* upstream region, we could not distinguish whether this region is part of a long non-coding RNA (lncRNA) with a start position further upstream or whether it is part of longer non-annotated *UTY* transcript. To confirm the expression of the other five newly discovered lncRNAs, we designed Y-specific primers for qPCR (**Supplementary Table 4**). We confirmed the expression of these regions human male fetal brain using independent samples from one male and one female embryos (E27 and E65 in **Table 1**). Additionally, we demonstrated expression of the orthologous regions in newborn male chimpanzee cerebral cortex indicating evolutionary conservation. The transcripts were absent in the female sample, except the *ARSEP1 downstream* region. Here, we detected expression also in a female chimpanzee sample, but not in the human female sample (**Table 1**). When we blasted these primer sequences against the chimpanzee genome, we noted that the primers bind with only one mismatch to a 143-bp region located 20,491bp at the 5’ side of the arylsulfatase D gene on the chimpanzee X-chromosome, most probably explaining the observed cross-amplification. All of the analyzed sequences had a higher expression in fetal male samples when compared to adult male samples, and the expression was higher in the adult male brain compared to adult testes (**Figure 5**).

### Comparison With Currently Published Datasets

To search for possible annotations of our six discovered lncRNAs, we consulted available lncRNA databases: NONCODE (Fang et al., 2018) and UCSC’s lincRNA RNA-Seq track (Trapnell et al., 2010; Cabili et al., 2011). As the annotations in UCSC’s lincRNA track were overlapping and derived from the same source as in NONCODE, we decided to focus on the annotations in the NONCODE database. The results are summarized in **Supplementary Table 3**. We could not find annotations that exactly matched our sequences regarding length, genomic position, and tissue expression. However, in all of the genomic regions covered by our lncRNAs, we found annotations for other lncRNA transcripts. These transcripts were mostly smaller, but in four cases, they were even longer than our lncRNA sequences. The NONCODE annotations are largely based on RNA-Seq data from Illumina’s Human BodyMap 2.0 project (Kapushesky et al., 2012), documenting expression in 16 different tissues from adult human. Of 19 partly overlapping sequences, 3 had their highest expression in the brain and 1 annotation (less than 1% overlap to *TTTY14*-DS) with highest expression in the brain was present on the opposite strand than our corresponding region. Compared to other tissues, the expression levels of the hits in the NONCODE database were rather low in brain. In conclusion, comparisons with currently available databases indicate that we have found six novel Y-lncs with restrictive high expression in the CNS before sexual maturation of the gonads.

## Discussion

We have studied sex-biased expression during early human development of human CNS, and we found that majority of genes that we detected were male-biased, accounting for approximately 4/5 of all differentially expressed genes (35 out of 43 genes—including the gametolog pairs). The low sensitivity of our analysis may have resulted in false negatives; thus, we may have missed genes with low expression and small differences. The largest biased group (23 genes) consisted of sex chromosome genes. Nine of the differentially expressed autosomal genes could be responsive to estrogen and/or androgens, and two are highly expressed in placenta (**Table 2**). This may reflect the age of the embryos which was close to the end of the first trimester. Amongst the male-biased genes were 15 previously known genes located in the male specific region of the Y-chromosome (MSY), 4 X-chromosome genes (*PAGE4*, *VGLL1, APLN*, and *CAPN6*), and 4 autosomal genes (*ANPT2, BHLHE40, MAFF*, and *SPON2*). Seven of the MSY genes (*PCDH11Y, EIF1AY, ZFY, DDX3Y, UTY, USP9Y, RPS4Y1*) were also found to be up-regulated in human NTRA-2 cells during differentiation (Vakilian et al., 2015). Only three female-biased genes have known gametologs on the Y-chromosome (*KDM5C, NLGN4X*, and *ZFX)*. When we estimated the gene dosage of the expression of X-chromosome genes with existing gametologs on the Y-chromosome, we found that, in 5 of 13 pairs, the expression was not balanced, resulting in a tendency toward higher total expression in males. One could have expected that X-/Y-gametologous pairs instead show female bias for the X-linked paralog in order to compensate for the expression of the Y-linked paralog (Jegalian and Page, 1998; Deng et al., 2014). However, two recent microarray studies on human brain during development (Kang et al., 2011) and adulthood (Trabzuni et al., 2013) also described a lack of compensation—in fact, the only gametologous gene with a significant female bias was *ZFX*.

A screen of publicly available databases for RNA-Seq data supported our presumption that many Y-chromosome genes are expressed at a very early time point in brain development. We found all of the Y-chromosome genes identified in the presented study (**Table 2**), to be expressed in brain samples from human male embryos at similar developmental stages (http://brainspan.org., Miller et al., 2014, and Human Developmental Biology Resource (HDBR) https://www.ebi.ac.uk/arrayexpress/experiments/E-MTAB-4840/, Papatheodorou et al., 2018) (**Table S5**). In brain samples of macaque, 8 of the 14 Y-chromosome genes (KDM5D, DDX3Y, EIF1AY, TXLNG2P, NLGN4Y, RPS4Y1, ZFY, TBL1Y) and one Y-chromosome gene that was not detected in human samples (RPS4Y2) were expressed at 60 days postconception (**Table S5** and Zhu et al., 2018).

When comparing our results with similar studies performed instead on adult-brain samples, we have noticed a remarkably higher number of sex-biased genes (about 10 fold) in adult samples (Trabzuni et al., 2013). But the contribution of sex-chromosome genes to the sex-specific expression signature is with 55% much higher in embryos compared to about 20% in adults (Trabzuni et al., 2013), underlining the importance of the sex chromosomes during early development. The mechanisms can be either direct and immediate caused by intrinsic X- or Y-chromosome gene activity or indirect—and in some cases even delayed—by transcriptional activation or epigenetic modification of autosomal genes. In contrast to our findings in humans, mouse studies using E10.5 brain samples (Dewing et al., 2003) and mouse embryonic stem cells (Werner et al., 2017) found that the vast majority of expression differences are not caused by genes located on the sex chromosomes but by autosomal genes. Thus, it is important to carefully consider the animal model and to be aware of possible evolutionary differences when interpreting rodent studies in a human context.

To further address the importance of the Y-chromosome, we searched for non-annotated genes and noncoding RNAs and found six expressed lncRNAs. For these six sequences, not exactly matching but partly overlapping annotations were found in the NONCODE database representing entries from the Illumina Human Body Map project. These data were retrieved from adult samples, and the expression in brain was rather low. Here, we could show a time- and tissue-specific expression suggesting an important function for these lncRNAs during early brain development.

Although some studies on lncRNAs excluded the Y-chromosome from their analysis (Lv et al., 2013; Barry et al., 2015), we found two publications that describe lncRNAs in the same regions as ours. A lncRNA downstream from the *TTTY14* gene (Y:21,093,942–21,237,874) was detected in a recent screen on various human cell lines and cancer specimens (Iyer et al., 2015). And a lncRNA at the *KDM5D* intergenic position (Y:20,519,948–20,524,433) was found in a screen using the human male cell line HepG2 to model coronary artery disease. The *KDM5D* intergenic lncRNA was ubiquitous expressed, but highest in the spleen and the heart, with subcellular localization in the nucleus (Molina et al., 2017). While the lncRNA downstream of *TTTY14* overlaps to 2/3 with the lncRNA we are describing (Y:21,033,832–21,213,188), the location of the lncRNA downstream of *KDM5D* does not overlap with our lncRNA in that region at all (Y:21,844,705–21,867,250).

LncRNAs exert their function on different levels: controlling transcription, exerting posttranscriptional and translational controls, controlling gene expression by epigenetic mechanisms, and directing of imprinting (Patil et al., 2014). Although it was initially thought that the significant amount of lncRNAs present on the Y-chromosome is mostly involved in testes development and spermatogenesis (Skaletsky et al., 2003), our analysis provides evidence that lncRNAs on the Y-chromosome may have important functions during early brain development and in the formation of gender-specific features.

All of our lncRNAs are conserved in the chimpanzee genome, and four regions (*KDM5D* downstream, *TTTY14* downstream, *UTY* downstream, and *TTTY15/USP9Y* intergenic) were detected by RNA-Seq in the brain of newborn male chimpanzee. QPCR indicated that even the additional two regions, *TXLNG2P upstream* and *ARSEP1 downstream*, were expressed in newborn male chimpanzee brain, indicating that the expression of all these long RNAs is conserved during primate evolution (Palazzo and Lee, 2015). A recent study investigating the conserved expression of lncRNA during human and macaque cortex development failed to detect corresponding regions, implicating that the lncRNAs detected in our study are only conserved among higher primates (He et al., 2014).

In conclusion, our data suggest an important role for the Y-chromosome in very early development of the CNS and our newly discovered lncRNAs share characteristics that suggest they might be functional, including tissue specificity, temporal restriction, polyadenylation, and conservation of expression in chimpanzee.

## Materials and Methods

### Human and Chimpanzee Embryonic CNS Samples and Sample Preparation

Three brain samples for each sex were obtained from human embryos between the 7th and 11th gestational weeks. The gestational age was estimated from the last menstrual period and from crown-rump length of the fetus measured by ultrasound. Samples were dissected after surgical terminations of pregnancies performed at the University Hospital in Uppsala, and after maternal written consent and approval from the regional Ethics Committee in Uppsala (2011/329). Surgical procedures occurred under general anesthesia (intravenous administration of 1 ml alfentanil 0.5 mg/ml and 15–20 ml propofol 10 mg/ml) and under supply of a mix of air and oxygen. Evacuating the uterine cavity was performed by a gynecologist with vacuum aspiration. Prior to snap-freezing, the fetal tissue was rinsed several times carefully in PBS to remove blood and non-fetal tissue. Brain tissue samples from one female and one male chimpanzee from Kolmården Zoo were collected and snap-frozen after autopsies. The female chimpanzee died at birth, the male chimpanzee died at 3 days of age.

Samples for RNA sequencing and real-time PCR were snap-frozen on dry ice and stored at −80°C until RNA extraction. To determine the sex of each embryo DNA was extracted from arm tissue using DNeasy^®^ Blood & Tissue Kit (Qiagen). PCR on DNA samples was done using male specific primers for STS sY14 (SRY) (5´-GAATATTCCCGCTCTCCGGA-3´, 5´-GCTGGTGCTCCATTCTTGAG-3). For information about all samples, see **Table 1**.

### Preparation of RNA and RNA-Seq Libraries From Total and poly(A) RNA

Frozen tissue samples (70–190 mg) were homogenized in 2-ml TRIzol^®^ Reagent (Ambion) using a Ultra-Torrax T25 homogenizator (Labortechnik). Total RNA was extracted RiboPure Kit (Ambion) according to manufacturers´ instructions. Poly(A) RNA was enriched from 1-μg total RNA using MicroPoly(A)Purist Kit (Ambion) according to the manufacturer’s instructions. The quantity and quality of the input RNA were controlled using a RNA 6000 Pico chip on a Bioanalyzer (Agilent Technologies), and only RIN values above 7 were used in the analysis.

cDNA library preparation was conducted at the Uppsala Genome Centre (SciLifeLab). Briefly, an rRNA depletion step was performed with 56 mg as input amount for all samples, using the RiboMinus Eukaryote Kit (Life Technologies). Whole-transcriptome libraries were then constructed using the SOLiD Total RNA-Seq Kit (rev B, July 2011, Life Technologies). Emulsion PCR was performed using the SOLiD EZ Bead System (Life Technologies), and the libraries were then sequenced on three lanes with the SOLiD 5500xl System (Life Technologies).

### Sequence Alignment and Data Analysis

After sequencing, all human reads where aligned using the LifeScope (version 2.5 Life Technologies) software using the hg19 version of the human genome. Reads where counted using the python script HTSeq (http://www.huber.embl.de/users/anders/HTSeq/doc/overview.html) (Anders et al., 2015) counting reads with a quality of 30 or higher to avoid reads mapping to more than one genomic location (Johansson et al., 2016) and using the default setting “union.” With this setting, HTSeq will only count reads falling into exonic regions of the gene, where no other genes overlap on the same strand. Most previous RNA-Seq analysis count reads with quality of 20 or higher (Sheng et al., 2017). Although these previous analysis were more sensitive than ours and have better chances of detecting larger number of genes, they have the problem of inflating results for gametologous pairs (due to high sequence identity between X/Y pairs). Furthermore, X-encoded genes with expressed pseudogenes (with high sequence identity to them) would be wrongly detected as escapees of X inactivation. Exonic regions for genes in the human genome were obtained from the iGenomes project (Illumina) (Quinlan and Hall, 2010). To calculate differential expression of genes between sequenced samples, the R library DESeq2 was used (Anders and Huber, 2010). Calculations of the expression in RPKM for each gene were performed using the RPKM function of the R library edgeR (Robinson et al., 2010). For Y-encoded genes, since females also had background levels due to misaligned repetitive elements, the same method used for X-genes and autosomal genes could be used for calculation of significance. For calculating differential gene expression, DESeq2 was used (Love et al., 2014). DESeq2 approximates the null distribution using a negative binomial model under the assumption that the two conditions have the same read abundance, in order to calculate the probability of genes having a differential expression between two samples. As count data are quite noisy when the expression levels are low, DESeq2 shrinks log-fold change estimates toward zero in a way so that the shrinkage is stronger when the information available for a gene is low (when counts are low, dispersion is high or where there are few degrees of freedom). These shrunken logarithmic fold changes and their standard errors are then used in the Wald test for differential expression. This procedure helps to circumvent the problem with extremely exaggerated fold changes for low counts. Correction for multiple testing was done by DESeq2 using the procedure of Benjamini and Hochberg.

### RT-qPCR Analysis

Primers were designed using the IDT OligoAnalyzer Tool (http://eu.idtdna.com/calc/analyzer), with the following parameters: Oligo 0.25 µM, Na+ 50.00 mM, Mg++ 1.00 mM, and dNTPs 1.00 mM. The NCBI’s Primer-BLAST tool was used to blast the primers against the human and chimpanzee genomes; selected pairs had 100% sequence identity with the homologous region in chimpanzee. Poly(A) RNA was reverse transcribed using a DyNAmo cDNA Synthesis Kit F-470L (Finnzymes) and the following reagents: 5 µl poly(A) RNA, 15 ng/µl random hexamers, 10 U M-MuLV RNase H– Reverse Transcriptase, 1 × RT buffer, and ddH_2_O, in a total reaction volume of 20 µl. Incubations were performed in a PTC-100 Peltier Thermal Cycler (MJ Research): 25°C, 10 min; 37°C, 45 min; and 85°C, 5 min. cDNA samples were subsequently diluted 1:40 in ddH_2_O. QPCR reactions contained 0.3 µM of each primer, 1×Power SYBR Green Master Mix (Applied Biosystems), 4-µl diluted cDNA sample, and ddH_2_O, in a total reaction volume of 25 µl. Thermal cycles were: 50°C; 2 min, 95°C; 10 min, 40 cycles: 95°C; 15 s, and 62°C, 1 min (for primer see **Supplementary Table 4**). To test for product specificity, a melting program was run subsequently to quantification. Expression was normalized to the geometric mean of *GAPDH*. RNA samples from human adult testes, human adult male brain, and human fetal brain were obtained from Takara Bio USA, Inc.

## Data Availability

The data has been made public at the Sequence Read Archive (SRA) (https://trace.ncbi.nlm.nih.gov/Traces/sra/sra.cgi?view=announcement) with the following identifiers: SRA: SRP093532 and BioProject: PRJNA347515.

## Ethics Statement

This study was carried out in accordance with the recommendations of the Swedish Ethical Review Authority. The protocol was approved by the Regional Ethics Committee in Uppsala (permit ID (2011/329). All donors at University Hospital in Uppsala gave written informed consent in accordance with the Declaration of Helsinki.

## Author Contributions

Performed experiments: MJ, PS, TA, and AZ. Analysed the data: MJ, PS, TA, JH, PP, and CP. Wrote the paper: MJ, EJ, CP, and PP with comments from all co-authors that have seen and approved the last version. Conceived the study and planned the experiments: MJ, PS, LF, and EJ. Contributed material and reagents: ED, LF, and EJ.

## Funding

This study was funded by the Swedish Research Foundation, grant name: Sex determination factors in the brain encoded in the Y-chromosome (Project number K2012-61X-22089-01-3). Salary support to JH was funded by the European Research Council ERC Starting Grant Agreement n. 282330 (to LF).

## Conflict of Interest Statement

The authors declare that the research was conducted in the absence of any commercial or financial relationships that could be construed as a potential conflict of interest.
